# Targeting P4HA1 Inhibits Colorectal Cancer Growth, Metastasis, and Tumor‐Associated Macrophage Infiltration via P4HA2‐PI3K‐AKT Pathway

**DOI:** 10.1002/iid3.70315

**Published:** 2025-12-30

**Authors:** Nanlin Cao, Yuan Li, Zhijie Chen, Zuliang Deng, Yangzhi Hu

**Affiliations:** ^1^ Department of General Surgery Affiliated Hospital of Xiangnan University Chenzhou P.R. China; ^2^ Nursing Department of Affiliated Hospital of Xiangnan University Chenzhou P.R. China; ^3^ Clinical College of Xiangnan University Chenzhou P.R. China; ^4^ Department of Gastrointestinal Surgery Affiliated Hospital of Xiangnan University Chenzhou P.R. China

**Keywords:** colorectal cancer, metastasis, P4HA1, P4HA2, tumor‐associated macrophages

## Abstract

**Background:**

Colorectal cancer (CRC) is a leading cause of cancer‐related mortality, which necessitates the exploration of novel therapeutic targets.

**Objective:**

This study aims to investigate the effects of Prolyl 4‐hydroxylase subunit alpha‐1 (P4HA1) inhibition on CRC tumor growth, metastasis, and tumor‐associated macrophage (TAM) infiltration.

**Methods:**

The association between P4HA1 expression and CRC progression as well as tumor immune infiltration was analyzed. In vitro experiments were performed to evaluate the effect of targeting P4HA1 on CRC cell proliferation and migration. The secretion of CCL2, CCL4, and CCL7 and recruitment of TAMs were detected after P4HA1 knockdown. Mechanistic studies were conducted to explore the interaction between P4HA1 and P4HA2 and its regulation on the PI3K‐AKT signaling pathway. In vivo experiments were also carried out to verify the effect of P4HA1 knockdown on CRC tumor growth, metastasis, and TAM infiltration polarization.

**Results:**

P4HA1 expression was found to be associated with CRC progression and tumor immune infiltration. Targeting P4HA1 significantly suppressed CRC cell proliferation and migration in vitro. Moreover, P4HA1 knockdown reduced the secretion of CCL2, CCL4, CCL7 and the recruitment of TAMs. Mechanistically, P4HA1 interacted with P4HA2, thereby disrupting the PI3K‐AKT signaling pathway which is crucial for CRC progression and TAMs recruitment. In vivo experiments confirmed that P4HA1 knockdown inhibited CRC tumor growth, metastasis, and TAM infiltration polarization.

**Conclusion:**

Our findings elucidate the importance of the P4HA1‐P4HA2‐PI3K‐AKT axis in CRC and identify P4HA1 as a promising therapeutic target to impede CRC growth and metastasis while altering the tumor immune landscape. This research provides a foundation for further investigations into P4HA1‐targeted therapies, which may improve clinical outcomes for patients with CRC.

## Graphical abstract

P4HA1 is associated with CRC progression and tumor immune infiltration; P4HA1 knockdown reduces CCL2, CCL4, CCL7 secretion and TAMs recruitment. P4HA1 knocking down inhibited CRC tumor growth, metastasis, and TAM infiltration polarization.

## 1 Introduction

Colorectal cancer (CRC) remains one of the leading causes of cancer‐related morbidity and mortality worldwide [1]. Research indicates that the 5‐year survival rate for patients with early‐stage CRC without metastasis exceeds 80%. In contrast, patients with distant metastasis have a markedly reduced 5‐year survival rate of only 10%–20% [2, 3]. Despite advancements in early detection and treatment strategies, the prognosis for advanced‐stage CRC remains poor, necessitating the exploration of novel therapeutic targets [4].

The prolyl 4‐hydroxylase (P4H) enzyme functions as a heterotetramer, consisting of three subtypes of the P4HA subunit (P4HA1, P4HA2, and P4HA3), which form α2β2 heterotetramers with P4HB [5]. These interactions result in the formation of P4H1, P4H2, and P4H3 holoenzymes, respectively. Notably, the β subunit of this enzyme complex occurs at higher concentrations than the α subunit [5]. The P4HA‐encoded α subunit contains the primary catalytic sites and is considered the rate‐limiting component for P4H enzymatic activity [5]. Prolyl 4‐hydroxylase subunit alpha 1 (P4HA1) has emerged as a pivotal enzyme in the regulation of collagen synthesis and subsequent remodeling of the extracellular matrix, processes that are crucial for tumor progression and metastasis [6, 7].

Recent studies have highlighted the role of P4HA1 in promoting tumor growth and metastasis across various cancer types, such as the P4HA1/HIF1 pathway, which is essential for the stemness of breast cancer cells [8]. For pancreatic cancer, the P4HA1‐HIF1α loop plays an important role in regulating glycolysis and oncogenic activity [9]. Under hypoxic conditions, both P4HA1 and HIF1α are upregulated; crucially, HIF1α acts functionally downstream of P4HA1, mediating its regulatory effects on human CRC cells via the Wnt signaling pathway [10]. Tumor‐associated macrophages (TAMs) are a significant component of this microenvironment, fostering an immunosuppressive environment that facilitates tumor growth and metastasis in CRC [11]. However, the role of P4HA1 in the tumor microenvironment still needs to be further explored.

This study investigates the role of P4HA1 in CRC, focusing specifically on its impact on tumor growth, metastasis, and TAM infiltration. Moreover, we further discovered a novel signaling pathway in which P4HA1 interacts with P4HA2, thereby activating the PI3K‐AKT pathway to promote CRC growth, metastasis, and TAM infiltration. Understanding this novel mechanism may pave the way for innovative treatments aimed at improving outcomes for CRC patients.

## 2 Materials and Methods

### 2.1 Patients and Samples

Paraffin‐embedded tumor samples from 90 CRC patients who underwent surgery at the Affiliated Hospital of Xiangnan University from February 2015 to July 2019 were retrospectively analyzed. The specimens of all patients were confirmed by the pathology department. All cases did not receive neoadjuvant radiotherapy and chemotherapy before the operation. Clinical data, clinical features, and follow‐up results were obtained. This scientific research was approved by the Ethics Committee of the Affiliated Hospital of Xiangnan University (K202303001).

### 2.2 Database Analysis

Gene Expression Profiling Interactive Analysis (GEPIA version 2) (http://gepia2.cancer-pku.cn/) was used to analyze the P4HA1 expression level in CRC [12]. The Kaplan–Meier Plotter (https://kmplot.com/analysis/) was used to analyze the prognostic value of P4HA1 in CRC data sets of the Gene Expression Omnibus database [13]. The correlation between P4HA1 expression levels and immune infiltration and related chemokine mRNA levels was analyzed in TIMER2 in colon adenocarcinoma (COAD) [14].

### 2.3 Cell Culture

The human colorectal adenocarcinoma cell lines HT‐29, SW620, and HCT116, normal human colonic epithelial cells NCM460, and THP‐1 cells were obtained from ATCC. Cells were cultured in Dulbecco's Modified Eagle Medium supplemented with 10% fetal bovine serum (FBS) and 1% penicillin‐streptomycin, and cells were maintained under identical conditions in a humidified atmosphere of 5% CO_2_ at 37°C. Cells were passaged every 2–3 days upon reaching 70%–80% confluence by detaching with trypsin‐EDTA. Cell line authentication was conducted via short tandem repeat profiling every 6 months.

### 2.4 Cell Counting Kit‐8 (CCK‐8) Assay

Cell viability was assessed using the CCK‐8 assay (Beyotime, C0038). HT‐29 and SW620 cells were seeded at a density of 1 × 10^3^ cells per well in 96‐well plates and allowed to adhere for 24, 48, and 72 h. Ten microliters of CCK‐8 solution was added to each well. The plates were then incubated at 37°C for 1 h, to allow for color development. The absorbance was measured at 450 nm using a microplate reader. Cell viability was calculated as a percentage relative to the control group. Experiments were performed in triplicate and repeated at least three times independently.

### 2.5 Colony‐Formation Assays

Colony‐formation assays were performed to evaluate the long‐term anchorage‐dependent growth of HT‐29 and SW620 cells. Cells were seeded at a density of 200 cells per well in 12‐well plates and allowed to grow for 14 days. Following the treatment period, colonies were fixed with 4% paraformaldehyde (PFA) solution for 15 min and subsequently stained with 0.1% crystal violet for 30 min. Data were expressed as the mean number of colonies ± standard deviation, with all experiments conducted in triplicate to ensure reliability.

### 2.6 Invasion, Migration, and TAM Recruitment Assays

Invasion and migration abilities of HT‐29 and SW620 cells were assessed using Transwell chamber assays. For the invasion assay, the upper chamber of a transwell was coated with Matrigel and then allowed to solidify. Cells were seeded at a density of 5 × 10⁴ cells in serum‐free medium in the upper chamber and allowed to adhere for 6 h. The lower chamber contained complete medium with 10% FBS. After 24 h, non‐invading cells in the upper chamber were removed with a cotton swab, while invading cells on the lower surface were fixed with a 4% PFA solution and stained with crystal violet. For the migration assay, the procedure was similar, except that Matrigel was omitted. Cells were again seeded in serum‐free medium and allowed to migrate toward the lower chamber for 12 h. THP‐1 cells and macrophages were treated with phorbol‐12‐myristic acid‐13‐acetate (PMA) for 24 h (#P8139, Sigma). Macrophages (2 × 10^5^) were resuspended in serum‐free protein medium and placed in the 8.0‐μm upper Transwell chamber, and 500 µL culture medium was added to the lower chamber. The migrated cells were counted under a microscope, and the results were expressed as the mean number of cells per field from three independent experiments.

### 2.7 Immunohistochemistry (IHC)

IHC was performed on paraffin‐embedded sections of clinical samples and mouse tumor tissues to evaluate protein expression. Tissue samples were deparaffinized in xylene and rehydrated through a series of graded alcohols. Antigen retrieval was achieved using heat‐induced epitope retrieval in citrate buffer (pH 6.0) for 20 min. Endogenous peroxidase activity was quenched with 3% hydrogen peroxide for 10 min. Sections were then blocked with BSA for 1 h at room temperature. Primary antibodies (anti‐P4HA1 antibody [Abcam, ab244400, 1:400]; rabbit anti‐CD68 mAb [Cell Signaling Technology, 76,437, 1:400]; rabbit anti‐CD163 mAb [Cell Signaling Technology, #93498, 1:400]; and rabbit anti‐CD74 [Cell Signaling Technology, #77274, 1:500]) were applied at optimized dilutions overnight at 4°C. After washing with phosphate‐buffered saline, sections were incubated with biotinylated secondary antibodies for 1 h at room temperature, followed by detection using an avidin‐biotin complex method (ABC kit). Staining was visualized using a DAB substrate, and sections were counterstained with hematoxylin. Images were captured using a light microscope, and protein expression levels were evaluated semiquantitatively by two independent pathologists. The immunohistochemical staining area was evaluated using Image‐Pro Plus 6.0, and expression was evaluated according to the average density.

### 2.8 Enzyme‐Linked Immunosorbent Assay

CCL2, CCL4, CCL7, CCL8, CXCL10 concentrations were measured using an ELISA kit (CCL2, ab179886, Abcam; CCL4, ab100597, Abcam; CCL7, ab193769, Abcam; CCL8, ab223856, Abcam; CXCL10, ab83700, Abcam) according to the manufacturers' instructions.

### 2.9 RT‐qPCR

Total RNA was extracted from cells using TRIzol reagent (Invitrogen, Carlsbad, CA, USA). cDNA was synthesized according to the manufacturer's protocol (Takara). RT‐qPCR was conducted using an Applied Biosystems instrument with a reaction volume of 20 µL (SYBR Green Real‐Time PCR Master Mix; Tsingke, Beijing, China). GAPDH was used as an internal control. All samples were tested in triplicate. The RT‐qPCR primers utilized in this study are listed in Supporting Information S2: Table 1.

### 2.10 Western‐Blot

Total protein was extracted from cells using RIPA total protein lysis buffer (Catalog # 89,900, Thermo Scientific, USA). The extracted proteins were separated by sodium dodecyl sulfate‐polyacrylamide gel electrophoresis and subsequently transferred to a 0.22‐µm PVDF membrane. To block non‐specific binding sites, the membrane was treated with 5% BSA at room temperature. Samples were then incubated with a primary antibody (anti‐P4HA1 antibody [Abcam, ab244400]; anti‐P4HA2 antibody [Abcam, ab211527]; PI3K/AKT signaling pathway [Abcam, ab283852]) overnight at 4°C, followed by a 1‐h incubation with a species‐appropriate HRP‐conjugated secondary antibody. Finally, immunoreactive proteins were detected using an ECL chemiluminescence detection kit. All quantitative Western blot data presented in the figures were normalized to β−actin.

### 2.11 CRC Cell Lines Knockdown and Overexpression

To investigate the effects of P4HA1 knockdown, shRNA lentiviruses targeting P4HA1 were employed. The specific shRNA sequences (Supporting Information S2: Table 2) were cloned into a lentiviral vector, and viral particles were produced in 293T cells using a standard packaging system. Target cells were transduced with the lentiviral particles in the presence of polybrene to enhance transduction efficiency. Following transduction, cells were selected with puromycin to establish stable P4HA1 knockdown lines. To assess the impact of P4HA1 overexpression in CRC cell lines, the full‐length P4HA1 cDNA was cloned into an expression vector. The vector was transfected into CRC cells using Lipofectamine 3000, following the manufacturer's protocol. Knockdown or overexpression efficiency was confirmed via Western blot analysis.

### 2.12 Co‐Immunoprecipitation (co‐IP)

To analyze the interaction between P4HA1 and P4HA2 in HT‐29 cells, a co‐IP assay was performed. HT‐29 cells were lysed in ice‐cold lysis buffer containing protease inhibitors. The cell lysates were pre‐cleared with protein A/G agarose beads and then incubated with an anti‐P4HA1 antibody overnight at 4°C. Protein A/G agarose beads were added to capture the immune complexes. After washing, the proteins were eluted and analyzed by Western blot using an anti‐P4HA2 antibody to detect interactions. The reverse co‐IP was conducted using an anti‐P4HA2 antibody to verify the findings.

### 2.13 Immunofluorescence Analysis

After 24 h of continuous exposure to 10%, 20%, and 30% sucrose solutions, five fixed malignant tumors were embedded in OCT and subsequently sliced into 5‐mm thick sections. This preparation was crucial for the subsequent immunostaining process, allowing for the effective analysis of the tumor samples. Following the slicing, the glass slides containing the tumor sections were incubated with a primary antibody at 4°C overnight. This step ensured that the primary antibody adequately bound to the target antigens within the tissue. Once this incubation was complete, the slides were then treated with a secondary antibody conjugated to Alexa Fluor dye at room temperature for 1 h. This dual‐antibody method heightened the visibility of the target proteins, facilitating more detailed observation. To further enhance the imaging process, DAPI was used for nuclear staining, which highlighted the nuclei of the cells and provided additional context for the observed structures. Finally, the sections were examined using a Zeiss LSM 800 confocal microscope.

### 2.14 Animal Experiments

The mouse orthotopic tumor model was established as described previously [15]. Luc‐labeled CRC cells were injected into the cecal serosa of BALB/c‐nu nude mice. The study included the negative control group and the shP4HA1 group. For tumor growth assessment, each group has five mice. For the orthotopic tumor model, each group had 8 mice. Six to 8 weeks post‐modeling, in vivo imaging was used to evaluate tumor metastasis to the liver in mice. The gross specimens of the mice, alongside colon tumors and liver metastases, were examined. All animal experiments were approved by the Animal Ethics Committee of Affiliated Hospital of Xiangnan University (DW‐2021‐015‐01).

### 2.15 Statistical Analysis

Statistical analysis was conducted using GraphPad Prism 9.0 (GraphPad Software, San Diego, California), and the data are presented as the mean ± SD (*X*). The differences between the two groups were evaluated using the two‐tailed unpaired *t*‐test or the Mann–Whitney test, and the differences among more than two groups were assessed using one‐way ANOVA and Tukey's post hoc test. A *p*‐value of < 0.05 was considered to indicate a significant difference. The Kaplan–Meier method was utilized to generate a survival curve, and the log‐rank test was employed for comparison.

## 3 Results

### 3.1 P4HA1 Expression Is Associated With CRC Progression and Tumor Immune Infiltration

**Figure 1:**
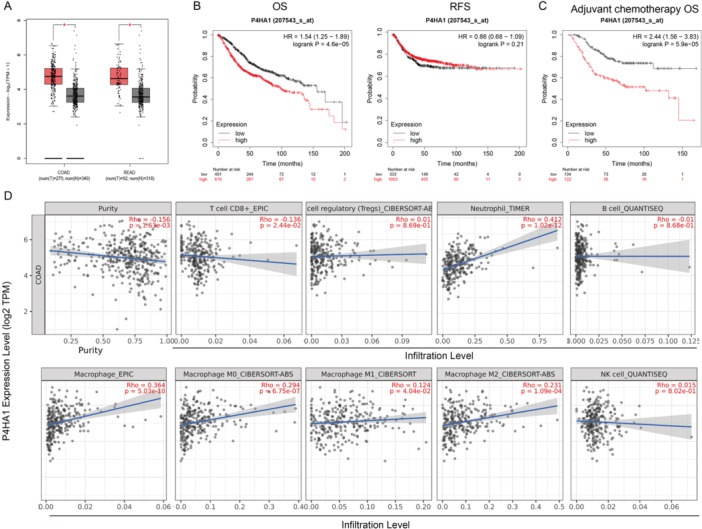
P4HA1 expression is associated with CRC prognosis and tumor immune infiltration. (A) GEPIA database was used to analyze the differential expression of P4HA1 between cancer and normal tissue in COAD (Tumor (T) *n* = 275, Normal (N) *n* = 349) and READ (Tumor (T) *n* = 92, Normal (N) *n* = 318). (B and C) Kaplan–Meier analysis showed P4HA1 expression prognosis in CRC patients. (D) The TIMER 2.0 database system was used to analyze the correlation between P4HA1 expression and CD8+ T cells, Tregs, neutrophils, B cells, macrophages, M0 macrophages, M1 macrophages, M2 macrophages, and NK cells in colon adenocarcinoma (COAD). Data represent mean ± SD, ns, no significant difference, *p < 0.05.

To clarify the role of P4HA1 in CRC progression and tumor immune microenvironment (TIM), we analyzed the P4HA1 expression level in the GEPIA database. As shown in Figure 1A, P4HA1 expression was significantly increased in COAD/rectum adenocarcinoma (READ) compared with normal colon tissue. Moreover, Kaplan–Meier analysis showed that high P4HA1 expression was associated with poor OS in CRC patients, especially for CRC patients with adjuvant chemotherapy (Figure 1B,C). Then we analyzed the relationship between P4HA1 expression and TIM in the TIMER database. High P4HA1 expression correlated with lower CD8+ T cells, higher neutrophil and macrophage infiltration (Figure 1D). Specifically, higher P4HA1 expression significantly correlated with M2 macrophage infiltration (Figure 1D) in CRC. These results revealed that P4HA1 plays an important role in CRC progression and tumor immune infiltration, especially in macrophage infiltration and polarization.

### 3.2 P4HA1 Associated Poor Prognosis and Macrophage Infiltration in CRC

**Figure 2:**
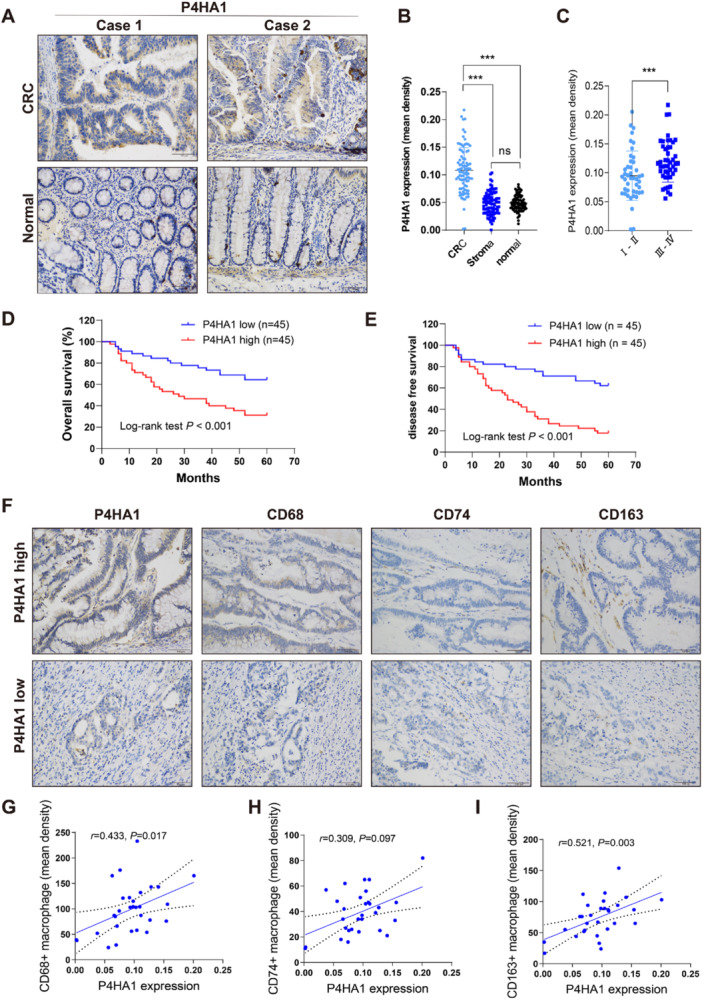
High P4HA1 expression is associated with tumor metastasis, poor prognosis, and TAM infiltration polarization in CRC. (A) Immunohistochemical staining was used to evaluate P4HA1 expression in CRC and adjacent normal tissues (100×); (B) P4HA1 was more highly expressed in cancer cells (*n* = 90) (one‐way ANOVA). (C) P4HA1 expression levels in colorectal cancer patients in Ⅰ–Ⅱ (*n* = 44) and Ⅲ–Ⅳ (*n* = 46) of TNM stage (two‐tailed unpaired *t*‐test). (D and E) Comparison of the overall survival (OS) rate and disease‐free‐survival (DFS) of CRC patients with different P4HA1 expression levels (Kaplan–Meier). (F) Levels of TAM (CD68), M1 (CD74), and M2 (CD163) macrophage infiltration of colorectal tumors with different P4HA1 expression levels (100×). (G–I) Correlation between P4HA1 expression level and density of TAM (CD68), M1 (CD74), and M2 (CD163) macrophage infiltration in CRC (*n* = 30) (Spearman correlation). Data represent mean ± SD, ns: no significant difference, ***p < 0.001.

First, IHC with 90 paired CRC and normal adjacent tissues revealed that P4HA1 was mainly expressed in tumor cells (Figure 2A), and significantly increased P4HA1 expression was noted in CRC tissues compared with tumor stroma or normal colon tissue (Figure 2B). To determine the role of P4HA1 in CRC, we analyzed the relationship between P4HA1 expression and clinicopathological features, and high P4HA1 expression was associated with TNM stage, especially in tumors with a high N and M stage (Figure 2C) (Table 1). Moreover, further survival analysis showed that high P4HA1 expression was associated with poor OS and DFS in CRC patients (*p* < 0.001) (Figure 2D,E). In addition, to verify that P4HA1 expression was correlated with TAM infiltration in CRC based on bioinformatics analysis, IHC was performed in serial sections of CRC tissues (Figure 2F). We found that high P4HA1 expression was correlated with TAM (CD68) (*r* = 0.433, *p* = 0.017) and M2 macrophage (CD163) (*r* = 0.521, *p* = 0.003) infiltration levels but not M1 macrophage (CD74) infiltration levels (*r* = 0.309, *p* = 0.097) (Figure 2G,I). Collectively, these data demonstrated that P4HA1 expression was correlated with poor prognosis, TAM infiltration, and M2 macrophage polarization in CRC.

**Table 1:** Correlations between P4HA1 expression and clinicopathological characteristics in CRC.

Parameters	No	P4HA1 expression		*p* value
		Low (*n* = 45)	High (*n* = 45)	
Age (years)				0.659
< 60	32	15	17	
≥ 60	58	30	28	
Gender				0.673
Male	46	24	22	
Female	44	21	23	
Differentiation gade				0.130
Well/moderate	55	31	24	
Poor/undifferentiated	35	14	21	
Tumor size (cm)				0.057
< 5	41	25	16	
≥ 5	49	20	29	
Tumor location				0.213
Right hemicolon	21	8	13	
Left hemicolon/Rectum	69	37	32	
Lymphovascular invasion				0.642
Abesent	64	31	33	
Present	26	14	12	
T stage				0.723
T1/T2	38	13	15	
T3/T4	52	22	30	
N stage				**< 0.001**
N0	44	30	14	
N1–N3	46	15	31	
M stage				**0.027**
M0	74	33	41	
M1	16	12	4	
TNM stage				**< 0.001**
Ⅰ/Ⅱ	44	30	14	
Ⅲ/Ⅳ	46	15	31	
MSI/MMR				0.694
pMMR/MSI‐L/MSS	83	41	42	
dMMR/MSI‐H	7	4	3	

*Note:* Bold values indicate statistical significance *p* < 0.05.

### 3.3 P4HA1 Knockdown Inhibits CRC Growth and Metastasis

**Figure 3:**
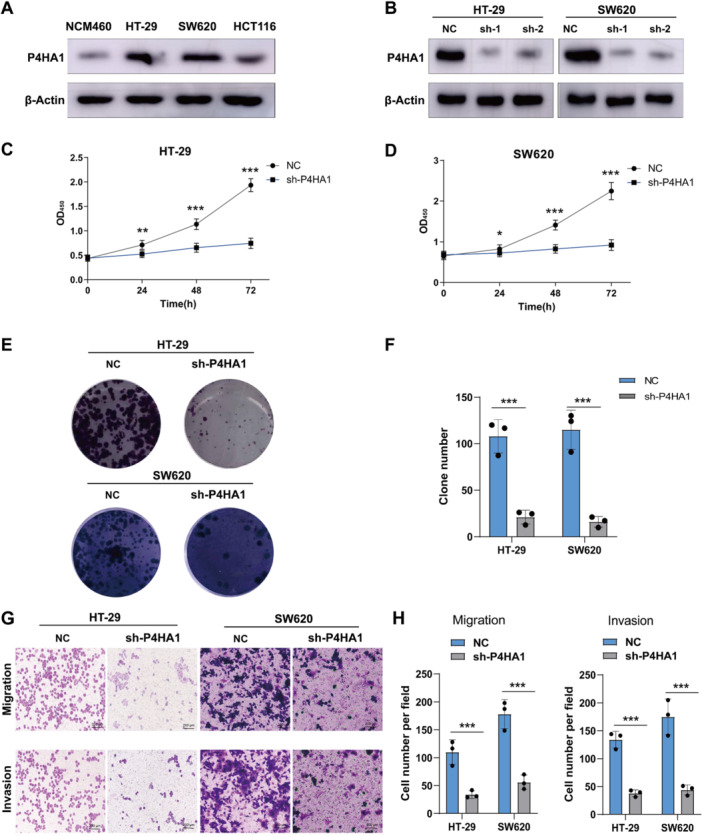
Knockdown of P4HA1 inhibits CRC cells' growth and metastasis. (A) Western‐blot analysis of P4HA1 expression of the NCM460, HT‐29, SW620, and HCT116 cells (*n* = 3 for each group) (two‐tailed unpaired *t*‐test). (B) P4HA1 knockdown and verification by Western blot. (C and D) Cell activity detection of P4HA1 knockdown in HT‐29 and SW620 cells by CCK‐8 assay. (E and F) Clonal formation assay for detecting cell growth after P4HA1 knockdown (*n* = 3 for each group) (two‐tailed unpaired *t*‐test). (G and H) Transwell assay for detecting cell migration and invasion after P4HA1 knockdown (100×) (*n* = 3 for each group) (two‐tailed unpaired *t*‐test). Data represent mean ± SD, ns: no significant difference, *p < 0.05, **p < 0.01, ***p < 0.001.

To further explore the function of P4HA1 in CRC growth and metastasis, first, up‐regulated expression of P4HA1 was found in CRC cell lines HT‐29, SW620, and HCT116 compared with normal intestinal mucosal epithelial cells NCM460. Moreover, we established a P4HA1 knockdown (sh‐P4HA1) cell model in HT‐29 and SW620 cells (Figure 3A,B). As shown in Figure 3C–F, P4HA1 knockdown markedly reduced cell viability and colony formation. In addition, P4HA1 knockdown also reduced HT‐29 and SW620 cells' invasion and migration (Figure 3H,I). Together, our results further indicated that P4HA1 promoted CRC cell growth and metastasis.

### 3.4 P4HA1 Knockdown Reduce CCL2, CCL4, CCL7 Secretion, and TAMs Recruitment in CRC

**Figure 4:**
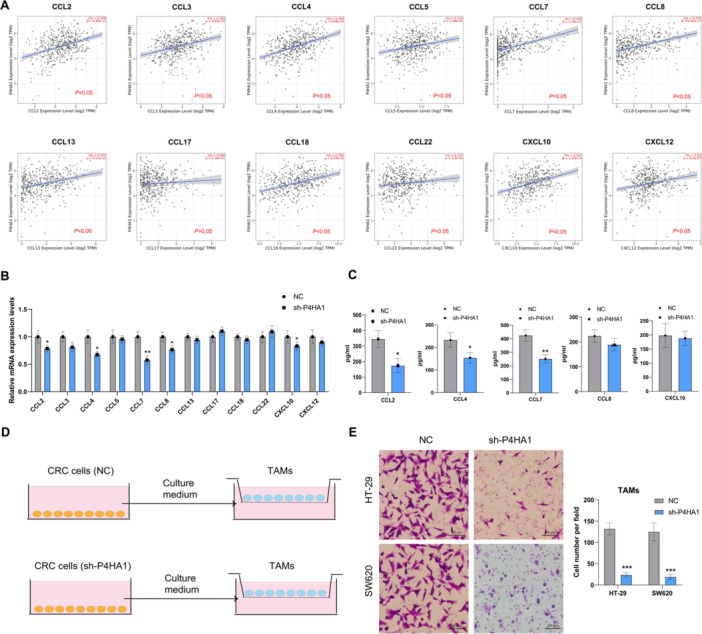
P4HA1 knockdown reduce CCL2, CCL4, CCL7 secretion and TAMs recruitment in CRC. (A) expression analysis between P4HA1 and related TAMs cytokine in CRC in the TIMER 2.0 database. (B) Rt‐qPCR was used to detect the related TAMs cytokine mRNA after P4HA1 knockdown in HT‐29 cells (*n* = 3) (two‐tailed unpaired *t*‐test). (C) ELISA assay was used to detect the CCL2, CCL4, CCL7, CCL8, and CXCL10 after P4HA1 knockdown in HT‐29 cells (*n* = 3) (two‐tailed unpaired *t*‐test). (D) Transwell model of CRC cells for recruiting TAMs. (E) Transwell assay for recruiting TAMs of P4HA1 knockdown in HT‐29 cells (100×) (*n* = 3) (two‐tailed unpaired *t*‐test). Data represent mean ± SD, ns: no significant difference, *p < 0.05, **p < 0.01, ***p < 0.001.

As we first found that P4HA1 expression was associated with TAMs infiltration, we hypothesized that P4HA1 regulates TAM‐related chemokines secretion in CRC. Therefore, we analyzed the correlation between P4HA1 and related chemokine expression in COAD. As shown in Figure 4A, P4HA1 expression significantly positive correlated with CCL2, CCL3, CCL4, CCL5, CCL7, CCL8, CCL13, CCL18, CCL22, CXCL10, CXCL12 (*p* < 0.05). Next, we detected the mRNA expression of these chemokines in P4HA1 knockdown HT‐29 cells. P4HA knockdown significantly reduced CCL2, CCL4, CCL7, CCL8, CXCL10 expression (Figure 4B). Does P4HA1 knockdown reduce these chemokine secretions? We also detected chemokine levels via ELISA. P4HA1 knockdown significantly reduced CCL2, CCL4, CCL7 secretion in HT‐29 cells (Figure 4C). To further clarify P4HA1‐mediated TAM recruitment, a transwell assay of THP‐1 cells was performed in the context of P4HA1 knockdown under co‐culture conditions (Figure 4D). As expected, P4HA1 knockdown significantly reduced TAM recruitment in HT‐29 or SW620 cells (Figure 4E). Therefore, targeting P4HA1 reduces CCL2, CCL4, and CCL7 secretion and TAMs recruitment in CRC.

### 3.5 P4HA1 Interacts P4HA2 and Activates PI3K‐AKT Pathway

**Figure 5:**
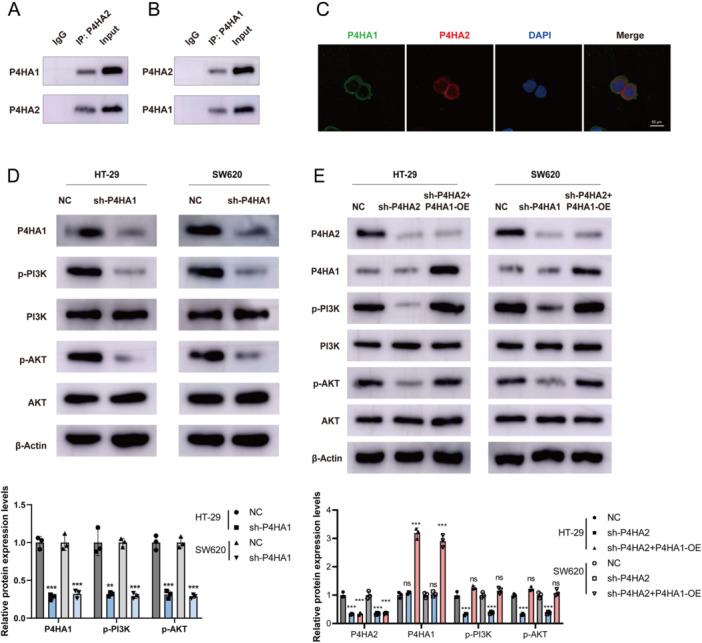
P4HA1 interacts P4HA2 and activates PI3K‐AKT pathway. (A and B) Co‐immunoprecipitation assay for detecting the interaction of P4HA1 and P4HA2. (C) Immunofluorescence assay for detecting interaction of P4HA1 and P4HA2 (400×). (D) Western blot for detecting PI3K‐AKT pathway activation after P4HA1 knockdown. (E) Western blot for PI3K‐AKT pathway change under P4HA2 knockdown with or without P4HA1 overexpression, **p < 0.01, ***p < 0.001.

To reveal the mechanism of P4HA1 in promoting CRC progression, we searched for the potential interaction target of P4HA1 in the HitPredict database. We found P4HA2 had the highest interaction score, indicating potential interaction with P4HA1. Therefore, we further detected the interaction between P4HA1 and P4HA2. As shown in Figure 5A,B, the interaction between P4HA1 and P4HA2 was confirmed via endogenous co‐IP in HT‐29 cells. Further immunofluorescence co‐localization assay also confirmed the interaction between P4HA1 and P4HA2 (Figure 5C). As P4HA2 regulates the PI3K/AKT signaling pathway in oral squamous cell carcinoma, we detect whether P4HA1 can regulate the PI3K/AKT signaling pathway via P4HA2 in CRC. First, knockdown of P4HA1 reduced PI3K and AKT phosphorylation in HT‐29 and SW620 cells (Figure 5D). Second, knockdown of P4HA2 also reduced PI3K and AKT phosphorylation; combined with P4HA1 overexpression, it rescued the PI3K and AKT phosphorylation levels in HT‐29 and SW620 cells (Figure 5E). Collectively, P4HA1 interacts with P4HA2 and then activates the PI3K‐AKT signaling pathway.

### 3.6 P4HA1 Promotes CRC Growth, Metastasis, and TAM Recruitment via P4HA2

**Figure 6:**
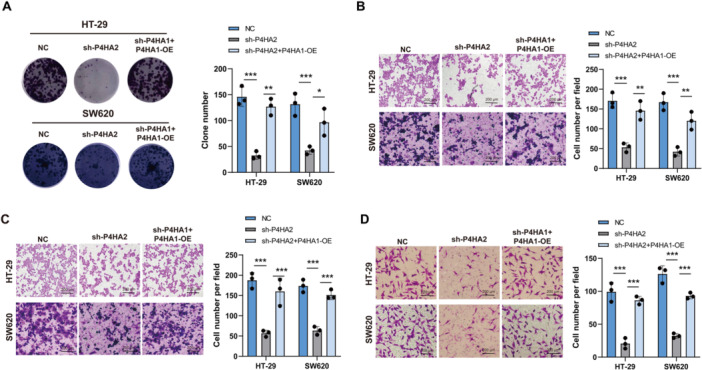
P4HA1 promotes cell growth, metastasis, and TAMs recruitment via P4HA2. (A) Clonal formation assay for detecting cell growth under P4HA2 knockdown with or without P4HA1 overexpression (*n* = 3 for each group) (one‐way ANOVA). (B and C) Transwell assay for detecting cell migration and invasion under P4HA2 knockdown with or without P4HA1 overexpression (*n* = 3 for each group) (one‐way ANOVA). (D) Transwell assay for recruiting TAMs in HT‐29 and SW620 cells (*n* = 3 for each group) (one‐way ANOVA). Data represent mean ± SD, ns: no significant difference, *p < 0.05, **p < 0.01, ***p < 0.001.

As P4HA2 is a critical downstream target of P4HA1, we further hypothesize that P4HA1 promotes CRC cell growth, metastasis, and TAM recruitment via P4HA2. We found knocking down P4HA2 inhibit HT‐29 and SW620 cells clonal formation, migration and invasion (Figure 6A–C). And P4HA1 overexpression can rescue the inhibitory effect of P4HA2 in HT‐29 and SW620 cells clonal formation, migration and invasion (Figure 6A–C). Moreover, knocking down P4HA2 also significantly reduces TAM recruitment, and combined with P4HA1 overexpression can increase TAM recruitment in HT‐29 and SW620 cells (Figure 6D). Therefore, these results confirm that P4HA1 promotes CRC growth, metastasis, and TAM recruitment via the P4HA2 pathway.

### 3.7 Targeting P4HA1 Inhibited Tumor Growth, Metastasis, and TAM Infiltration Polarization

**Figure 7:**
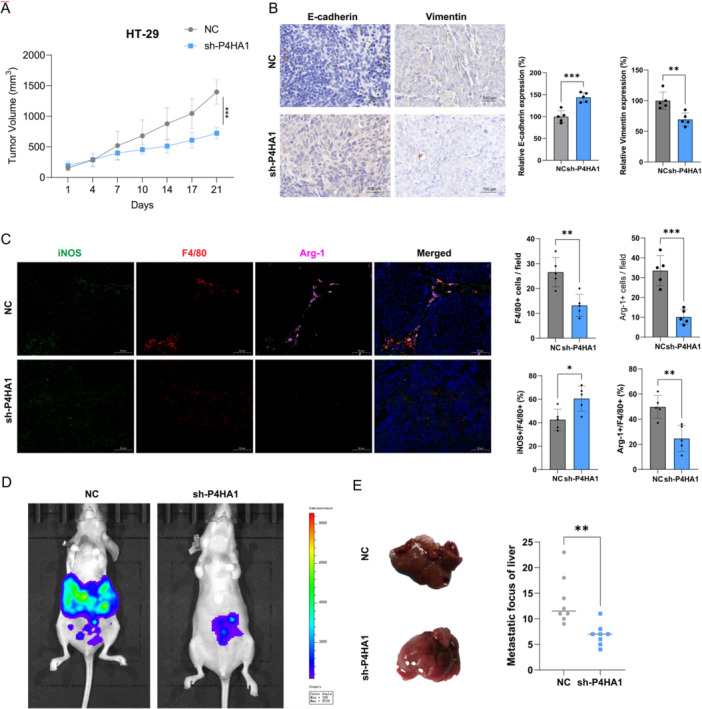
P4HA1 knockdown in CRC cells inhibits tumor growth, metastasis, TAM infiltration, and M2 polarization. (A) P4HA1 knockdown inhibits HT‐29 growth via xenograft model in nude mice (*n* = 5 for each group) (two‐tailed unpaired *t*‐test). (B) IHC analysis of E‐cadherin and Vimentin expression in P4HA1 knockdown xenograft tumor (100×) (*n* = 5 for each group) (two‐tailed unpaired *t*‐test). (C) The silver staining method was used to detect phagocyte (F4/80), M1 (iNOS), and M2 (Arg‐1) invasion in HT‐29 tumors after P4HA1 knockdown (100×) (*n* = 5 for each group) (two‐tailed unpaired *t*‐test). (D) A Luc‐HT‐29 orthotopic liver metastasis mouse model was used to evaluate the effect of P4HA1 knockdown on CRC somatic cells in vivo. (E) Representative images of liver tumors from different groups of mice (*n* = 5 for each group) (two‐tailed unpaired *t*‐test). Data represent mean ± SD, ns: no significant difference, *p < 0.05, **p < 0.01, ***p < 0.001.

Finally, we aim to explore the role of P4HA1 in vivo. First, knocking down P4HA1 significantly inhibited tumor growth in the HT‐29 xenograft model in nude mice (Figure 7A). Moreover, knocking down P4HA1 also reduced the increase in E‐cadherin expression and the decrease in Vimentin expression in tumor tissue (Figure 7B). And P4HA1 knocking did not impact tumor weight (Supporting Information S1: Figure 1) and main organs (Supporting Information S1: Figure 2) of mice. In addition, targeting P4HA1 also reduced the infiltration of macrophages (F4/80 +) and M2‐type (Arg +) macrophages, as well as M2‐type (F4/80+ Arg +) macrophages ratio and the increased infiltration ratio of M1‐type (F4/80+ iNOS +) macrophages (Figure 7C). Finally, targeting P4HA1 reduced the number of CRC liver metastases in the orthotopic liver metastasis model (Figure 7D,E). Collectively, these data suggest that targeting P4HA1 can inhibit CRC growth, metastasis, and TAM infiltration and polarization.

## 4 Discussion

In our study, we demonstrated that targeting P4HA1 significantly inhibits CRC growth, metastasis, and TAM infiltration, primarily through the modulation of the P4HA2‐PI3K‐AKT signaling pathway. These findings highlight the critical roles of P4HA1 and its associated pathways in CRC initiation and progression.

P4HA1, a key enzyme involved in collagen biosynthesis, has been previously implicated in various cancers [15, 16]. Our results corroborate previous studies [17] indicating that P4HA1 expression is upregulated in CRC tissues compared to adjacent normal tissues, and confirm that high P4HA1 expression correlates with poor prognosis in CRC. Elevated levels of P4HA1 may facilitate tumorigenic processes by enhancing collagen deposition, which, in turn, promotes tumor cell invasion and metastasis. By targeting P4HA1, we observed a marked reduction in cell growth and migratory capabilities, suggesting its potential as a therapeutic target in CRC. However, the relationship between P4HA1 and TME remains poorly reported. We first analyzed the P4HA1 expression and the tumor immune infiltration in CRC. And found high P4HA1 expression correlated with a variety of immune cell infiltration in CRC. Our clinical samples also further confirm the P4HA1‐associated poor prognosis and macrophage infiltration.

Tumor microenvironment dynamics, particularly the infiltration of TAMs, are essential for CRC progression [18]. Our results indicate that inhibiting P4HA1 not only directly impacts cancer cells but also modulates the immune landscape by reducing TAM infiltration. The reduction of TAMs may be attributed to the altered inflammatory microenvironment following the disruption of the P4HA2‐PI3K‐AKT signaling axis. Macrophages often promote tumor growth through various mechanisms, including the secretion of pro‐inflammatory cytokines that support tumor progression [19]. Thus, targeting P4HA1 could indirectly lessen the supportive role of TAMs in CRC.

For the PAHA1 mechanism for CRC progression, a previous study indicates that P4HA1 is a new regulator of the HIF‐1 pathway [20]. Moreover, P4HA1 can also activate HMGCS1 to promote nasopharyngeal carcinoma progression [21]. In our study, we found that P4HA1 interacts and activates P4HA2, then regulates the downstream PI3K‐AKT signaling pathway, which plays a pivotal role in cellular proliferation, survival, and chemokine secretion. As previous studies report, increased expression of P4HA2 was observed in various malignant tumors, including breast cancer, cervical cancer, and hepatocellular carcinoma [22, 23]. Our data show that the inhibition of P4HA1 leads to a downregulation of P4HA2 and subsequent inhibition of the PI3K‐AKT pathway, which is crucial for CRC cell growth and survival. This is consistent with findings by other researchers, who have identified aberrant activation of the PI3K‐AKT pathway as a significant contributor to CRC progression. In addition, targeting P4HA1 reduces CCL2, CCL4, and CCL7 secretion and TAMs recruitment in CRC. By reining in this pathway, targeting P4HA1 provides a novel mechanism to curb CRC development and progression.

The targeting of P4HA1 presents a promising therapeutic avenue for CRC. Current treatment strategies often involve a multi‐modal approach, including chemotherapy and targeted therapies. However, resistance to these therapies is a common challenge. Our findings suggest that P4HA1 inhibition may enhance the efficacy of existing therapies by targeting the molecular underpinnings of tumor growth and immune modulation. Future studies should explore the potential synergistic effects of combining P4HA1 inhibition with established treatment regimens. While our findings highlight P4HA1's therapeutic promise, potential off‐target effects warrant consideration. As P4HA1 catalyzes proline hydroxylation in collagen biosynthesis, its systemic inhibition could disrupt collagen maturation in normal tissues, potentially affecting wound healing or vascular integrity. Future studies should characterize isoform‐specific inhibitors to minimize cross‐reactivity with P4HA2/P4HA3 and evaluate tissue‐specific toxicity profiles in preclinical models.

While our findings are promising, there are certain limitations that warrant consideration. First, the study primarily relies on in vitro and animal models. Future clinical trials are necessary to validate the efficacy and safety of P4HA1 inhibitors in human populations. Second, the intricacies of the tumor microenvironment in CRC are complex, involving various cell types and signaling pathways. A more thorough exploration of these interactions could yield deeper insights into CRC biology and treatment response.

In conclusion, our research provides substantial evidence that targeting P4HA1 effectively inhibits CRC growth and metastasis, as well as TAM infiltration. Through the P4HA2‐PI3K‐AKT signaling pathway, P4HA1 emerges as a potential therapeutic target, offering a novel strategy for CRC treatment.

## Author Contributions

**Nanlin Cao:** conceptualization, methodology, writing – original draft. **Yuan Li:** formal analysis, methodology. **Zhijie Chen:** data curation. **Zuliang Deng:** investigation. **Yangzhi Hu:** conceptualization, funding acquisition, writing – review and editing.

## Conflicts of Interest

The authors declare no conflicts of interest.

## Supporting information


**Supplementary Figure 1:** Tumor weight comparison between NC and sh‐P4H41 group in HT‐29 bearing mice model. **Supplementary Figure 2:** Hematoxylin and eosin (H&E) staining images of main organs of mice in NC and sh‐P4H41 group in HT‐29 bearing mice model.

**Supplementary Table 1:** The qRT‐PCR primers for this study. **Supplementary Table 2:** The shRNA sequence for this study.

**Supplemental Table 3:** the potential interaction target of P4HA1.

## Data Availability

The data that support the findings of this study are available on request from the corresponding author.
