# Supplementary material for: Targeting P4HA1 Inhibits Colorectal Cancer Growth, Metastasis, and Tumor‐Associated Macrophage Infiltration via P4HA2‐PI3K‐AKT Pathway
Source: Immun Inflamm Dis. 2025 Dec 30;13(12):e70315. doi: 10.1002/iid3.70315 (PMC12753198; doi:10.1002/iid3.70315)
Supplement: Supplementary file 1 — Supplementary Figure 1: Tumor weight comparison between NC and sh‐P4H41 group in HT‐29 bearing mice model. Supplementary Figure 2: Hematoxylin and eosin (H&E) staining images of main organs of mice in NC and sh‐P4H41 group in HT‐29 bearing mice model. [file IID3-13-e70315-s003.docx]

Supplementary Figure 1. Tumor weight comparison between NC and sh-P4H41 group in HT-29 bearing mice model. Data represent mean ± SD, ns: no significant difference


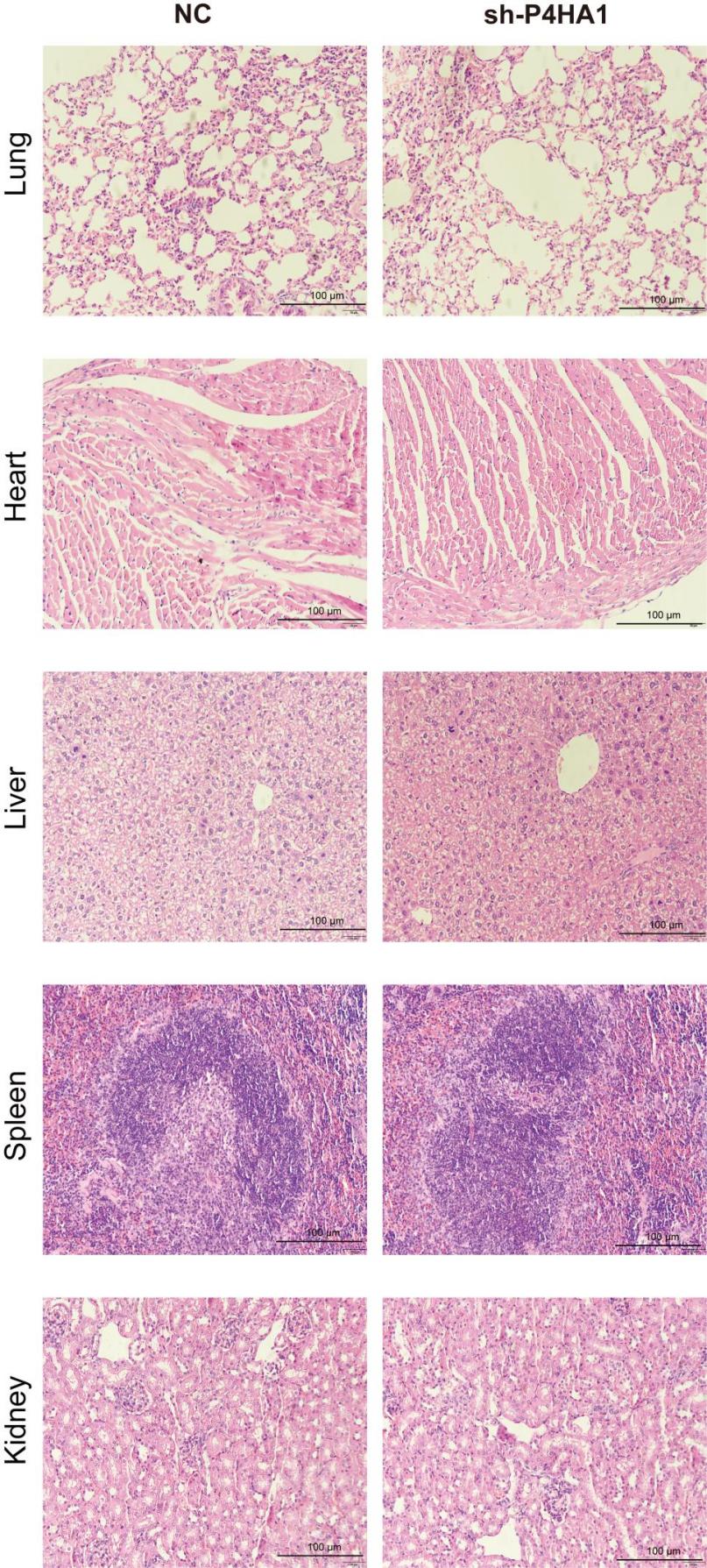


Supplementary Figure 2. Hematoxylin and eosin (H&E) staining images of main organs of mice in NC and sh-P4H41 group in HT-29 bearing mice model.
