# Supplementary material for: Targeting P4HA1 Inhibits Colorectal Cancer Growth, Metastasis, and Tumor‐Associated Macrophage Infiltration via P4HA2‐PI3K‐AKT Pathway
Source: Immun Inflamm Dis. 2025 Dec 30;13(12):e70315. doi: 10.1002/iid3.70315 (PMC12753198; doi:10.1002/iid3.70315)
Supplement: Supplementary file 2 — Supplementary Table 1: The qRT‐PCR primers for this study. Supplementary Table 2: The shRNA sequence for this study. [file IID3-13-e70315-s001.docx]

**Supplementary Table 1: The qRT-PCR primers for this study**

| **Gene** |  | **Primer** |
| --- | --- | --- |
| CCL2 | Forward | 5'-CAGCCAGATGCAATCAATGCC-3' |
|  | Reversed | 5'-TGGAATCCTGAACCCACTTCT-3' |
| CCL3 | Forward | 5'-AGTTCTCTGCATCACTTGCTG-3' |
|  | Reversed | 5'-CGGCTTCGCTTGGTTAGGAA-3' |
| CCL4 | Forward | 5'-CTGTGCTGATCCCAGTGAATC-3' |
|  | Reversed | 5'-TCAGTTCAGTTCCAGGTCATACA-3' |
| CCL5 | Forward | 5'-CCAGCAGTCGTCTTTGTCAC-3' |
|  | Reversed | 5'-CTCTGGGTTGGCACACACTT-3' |
| CCL7 | Forward | 5'-ACAGAAGGACCACCAGTAGCCA-3' |
|  | Reversed | 5'-GGTGCTTCATAAAGTCCTGGACC-3' |
| CCL8 | Forward | 5'-TGGAGAGCTACACAAGAATCACC-3' |
|  | Reversed | 5'-TGGTCCAGATGCTTCATGGAA-3' |
| CCL13 | Forward | 5'-CTCAACGTCCCATCTACTTGC-3' |
|  | Reversed | 5'-TCTTCAGGGTGTGAGCTTTCC-3' |
| CCL17 | Forward | 5'-TTCTCTGCAGCACATCCACGCA-3' |
|  | Reversed | 5'-CTGGAGCAGTCCTCAGATGTCT-3' |
| CCL18 | Forward | 5'-GTTGACTATTCTGAAACCAGCCC-3' |
|  | Reversed | 5'-GTCGCTGATGTATTTCTGGACCC-3' |
| CCL22 | Forward | 5'-ATCGCCTACAGACTGCACTC-3' |
|  | Reversed | 5'-GACGGTAACGGACGTAATCAC-3' |
| CXCL10 | Forward | 5'-GTGGCATTCAAGGAGTACCTC-3' |
|  | Reversed | 5'-TGATGGCCTTCGATTCTGGATT-3' |
| CXCL12 | Forward | 5'-ATTCTCAACACTCCAAACTGTGC-3' |
|  | Reversed | 5'-ACTTTAGCTTCGGGTCAATGC-3' |
| GAPDH | Forward | 5'-TCGGAGTCAACGGATTTGGT-3' |
|  | Reversed | 5'-TTCCCGTTCTCAGCCTTGAC-3' |

**Supplementary Table 2: The shRNA sequence for this study**

| **Gene name** | **Catalog No** | **Sequence** |
| --- | --- | --- |
| P4HA1 | shRNA-1 | GGAAUUACAGGUAGCAAAU |
| P4HA1 | shRNA-2 | GAUAAAGUCUCUGUUCUAG |
